# Effect of epigenetic treatment on SST_2_ expression in neuroendocrine tumour patients

**DOI:** 10.1002/ctm2.957

**Published:** 2022-07-22

**Authors:** Julie Refardt, Maria J. Klomp, Peter M. van Koetsveld, Fadime Dogan, Mark Konijnenberg, Tessa Brabander, Richard A. Feelders, Wouter W. de Herder, Leo J. Hofland, Johannes Hofland

**Affiliations:** ^1^ ENETS Center of Excellence, Department of Internal Medicine, Section of Endocrinology Erasmus Medical Center Rotterdam The Netherlands; ^2^ ENETS Center of Excellence, Department of Endocrinology University Hospital Basel Basel Switzerland; ^3^ ENETS Center of Excellence, Department of Radiology & Nuclear Medicine Erasmus Medical Center Rotterdam The Netherlands

Dear Editor,

Several preclinical studies have uncovered that epigenetic drugs can upregulate somatostatin receptor subtype 2 (SST_2_) expression in neuroendocrine tumour (NET) models,^1^, ^2^ which could be of eminent importance for NET patients with low tumoural SST expression. In a prospective clinical proof‐of‐concept trial involving nine advanced NET patients with low SST expression, we were able to show that epigenetic treatment with the histone deacetylase (HDAC) inhibitor valproic acid and the DNA methyltransferase (DNMT) inhibitor hydralazine did not lead to an increase in tumour‐uptake of ^68^Ga‐DOTATATE, contradicting the in vitro data.

A prerequisite for the treatment of advanced NETs with (radiolabelled) somatostatin analogues (SSA) is the expression of SST_2_ on the tumour cell surface, providing rationale for the inferior outcome in patients with low uptake on functional SST imaging.^3^ Several previous in vitro studies and one in vivo study achieved stimulation of SST_2_ expression levels and binding of SSAs by increasing histone acetylation levels and reducing DNA methylation of the SST_2_ gene promoter region in NET cells by epigenetic drugs.^1^, ^2^, ^4^ Despite these promising results, there are only data from one study showing limited increase of ^68^Ga‐DOTATOC uptake by HDAC inhibitor vorinostat in five NET patients already expressing SST at baseline.^5^

In the present study, which was approved by the Ethics Committee of the Erasmus Medical Center Rotterdam and registered at the Netherlands Trial Register (NL7726), nine patients with advanced NETs (Table 1) and low SST expression at baseline on ^68^Ga‐DOTATATE/PET (Table 2), defined as tumour uptake below or equal to the physiological uptake in the liver, were included and provided written informed consent. Patients were treated for 14 days simultaneously with the HDAC inhibitor valproic acid (30‐mg/kg body weight/day, max. 3000 mg/day) and the DNMT inhibitor hydralazine (150 mg/day). One week after start of treatment, valproic acid dosage wasadjusted to target a serum concentration of 75–120 μg/ml.^6^ Hydralazine dosage remained unchanged unless adjusted for tolerability. Treatment effect was evaluated after 2 weeks by the change in ^68^Ga‐DOTATATE uptake on PET/CT. The last two patients (lung NET, rectum NET) completed the trial without hydralazine due to emerging insights from the in vitro studies, which were performed simultaneously in three human NET cell lines BON‐1 (pancreatic NET), GOT1 (small intestinal NET) and NCI‐H727 (lung NET). Here, effects of valproic acid sodium salt and hydralazine on SST_2_ mRNA and protein levels as well as ^111^In‐DOTATATE uptake were assessed (details in the Supplementary Appendix).

**TABLE 1:** Baseline characteristics of the neuroendocrine tumour patients included in the clinical trial. Values are shown as median (interquartile range [IQR]) or number (%)

**Patient characteristics**	**Total** (*n* = 9)
Age, years (IQR)	67 (54, 75)
Sex (male), *n* (%)	5 (56)
**Origin**	
Pancreas NET, *n* (%)	2 (22)
Small intestinal NET, *n* (%)	1 (11)
Lung NET, *n* (%)	4 (44)
Rectum NET, *n* (%)	1 (11)
Thymus NET, *n* (%)	1 (11)
**Metastases**	
Lymph nodes, *n* (%)	9 (100)
Liver, *n* (%)	5 (56)
Mesenterial, *n* (%)	1 (11)
Bone, *n* (%)	3 (33)
Lung, *n* (%)	1 (11)
Other, *n* (%)	4 (44)
**Ki67 index**	
0%–2%, *n* (%)	3 (33)
5%–10%, *n* (%)	4 (44)
30%	1 (11)
Unknown	1 (11)
**Grading**	
G1, *n* (%)	4 (44)
G2, *n* (%)	4 (44)
G3, *n* (%)	1 (11)
**Previous treatments**	
Surgery, *n* (%)	3 (33)
Somatostatin analogue, *n* (%)	2 (22)
Chemotherapy, *n* (%)	1 (11)
Other, *n* (%)	3 (33)

Abbreviations: Bpm, beats per minute; *n*, number; NET, neuroendocrine tumour; IQR, interquartile range; SUV, standard uptake values.

**TABLE 2:** Change in study parameters of neuroendocrine tumour patients at baseline and after 1 and 2 weeks of epigenetic treatment

**Clinical parameters**	**Baseline**	**Week 1**	**Week 2**	***p* Value**
Weight, kg (IQR)	76 (68, 86)	77 (68, 88)	77 (69, 88)	.05
Blood pressure systolic, mmHg (IQR)	147 (130, 155)	139 (129, 151)	135 (126, 148)	.14
Heart rate, bpm (IQR)	69 (62, 81)	77 (67, 109)	76 (65, 96)	.34
**Laboratory parameters**				
Haemoglobin, mmol/L (IQR)	8.5 (8.1, 9.2)	8.5 (7.7, 9.2)	8.1 (7.6, 8.6)	.05
Thrombocytes, ×10^9^/L (IQR)	247 (195, 282)	233 (173, 255)	177 (148, 271)	.11
Creatinine, umol/L (IQR)	73 (58, 90)	74 (54, 86)	76 (56, 89)	.72
ASAT, U/L (IQR)	27 (23, 32)	23 (21, 30)	28 (24, 36)	1
ALAT, U/L (IQR)	26 (17, 35)	17 (16, 25)	21 (13, 26)	.09
GGT, U/L (IQR)	65 (19, 98)	46 (19, 82)	48 (18, 115)	.16
Valproic acid drug level, μg/ml (IQR)	NA	102 (84, 126)	95 (90, 117)	NA
**Study medication**				
Valproic acid dosage, mg/day (IQR) (*n* = 9)	NA	2300 (1900, 2500)	1900 (1763, 2000)	NA
Hydralazine dosage, mg/day (IQR) (*n* = 7)	NA	150 (150, 150)	150 (100, 150)	NA
**Tumour uptake of ^68^Ga‐DOTATATE**				
None, *n* (%)	6 (67)		6 (67)	1
Below liver, *n* (%)	3 (33)		3 (33)	1
**Peak uptake**				
Primary tumour, SUV (IQR)(*n* = 6)	8.1 (3.0, 11.4)		6.8 (2.8, 9.9)	.17
Lymph node metastases, SUV (IQR) (*n* = 5)	4.8 (3.1, 9.0)		5.8 (2.6, 7.8)	.35
Liver metastases, SUV (IQR) (*n* = 5)	7.5 (5.0, 7.9)		7.3 (4.5, 8.4)	.29
Bone metastases, SUV (IQR) (*n* = 4)	4.1 (2.6, 5.1)		4.2 (2.7, 5.2)	.47
Intestinal metastases, SUV (IQR) (*n* = 2)	9 (7.5, 10.5)		8.7 (6.7, 10.6)	.67
Skin metastases, SUV (IQR) (*n* = 1)	3.5		3.7	NA
Liver, SUV (IQR)	10.5 (8.3, 12.6)		10.7 (8.3, 12.3)	.95
Kidneys, SUV (IQR)	16.3 (14.3, 19.2)		20.7 (16.1, 26.0)	**.02**
Spleen, SUV (IQR)	25.9 (22.7, 32.7)		27.8 (22.0, 31.9)	.68

*Note*: Values are shown as median (IQR) or number (%) in nine patients, unless otherwise indicated. Bold writing signifies significance.

Abbreviations: *n*, number; NA, not applicable; IQR, interquartile range; SUV, standard uptake values.

At the end of the 2‐week epigenetic treatment period, none of the NET patients had an increase in ^68^Ga‐DOTATATE uptake grade (Table 2). No change in median ^68^Ga‐DOTATATE uptake in any NET sites was observed, and there was even a tendency for reduced uptake in primary tumours (Figures 1 and S1). These findings were independent of tumour aetiology, metastatic location or drug treatment. Meanwhile, a significant median (IQR) increase of 27% (4.1, 46.4) in uptake was observed in the kidneys, *p* = .02, independent of the study medication. A limitation of our study is the restricted patient number, but given the lack of effects in any of the patients with different NET origins, this protocol is unlikely to affect tumoural SST_2_ expression in vivo. All patients reported known side effects of the study medication (details in the Supplementary Appendix), and no serious adverse events occurred during the study.

**FIGURE 1:**
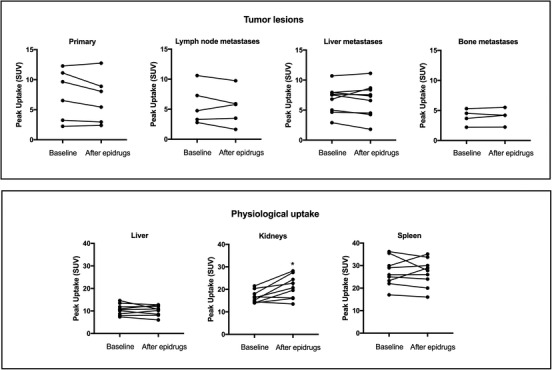
Change in peak uptake of ^68^Ga‐DOTATATE on PET/CT at baseline and after 2‐week epigenetic treatment in patients with neuroendocrine tumours with low somatostatin receptor expression. The upper panel showing changes in tumour lesions, the lower panel showing changes in physiological uptake. Patients were prepared according to our local protocol, which includes the drinking of 1 L of water in 2 h before injection. Imaging was performed from scull base to thighs after median (interquartile range [IQR]) 60 min (59–65) injection with an activity of 118‐MBq (103–121) ^68^Ga‐DOTATATE. For each patient, at least two tumour target lesions, including the primary if applicable, were defined on the initial ^68^Ga‐DOTATOC PET/CT. Peak standard uptake value (SUV) was calculated for every lesion as well as for the liver, kidneys and spleen. *p < .05 according to Wilcoxon signed‐rank test

In all cell lines tested, treatment with valproic acid led to a significant increase of SST_2_ mRNA levels and ^111^In‐DOTATATE uptake, *p* < .001, respectively (Figure 2). An increase in the SST_2_ staining intensity per cell was observed in BON‐1 and NCI‐H727 cells (*p* < .01), but not in GOT1 cells, possibly due to the high baseline SST_2_ expression levels (Figure S2). Meanwhile, an increase in SST_2_ mRNA levels was seen only for the stronger hydralazine dose in BON‐1 cells (*p* < .001) and GOT1 cells (*p* < .05), but hydralazine decreased mean (SD) SST_2_ mRNA expression levels in NCI‐H727 cells by 15% (13), *p* < .05. No changes in SST_2_ protein expression and ^111^In‐DOTATATE uptake were seen following incubation with hydralazine in all cell lines. The combined treatment of valproic acid with the stronger hydralazine dosage led to an additional mean (SD) increase in SST_2_ mRNA expression levels in BON‐1 cells of 120% (72), *p* < .001, whereas no additional effect was seen for GOT1 cells, and even an inhibitory mean effect (SD) of 73% (34), *p* < .001, was observed in NCI‐H727. No synergistic or antagonistic effect on ^111^In‐DOTATATE uptake and SST_2_ staining intensity per cell was observed for the combined treatment.

**FIGURE 2:**
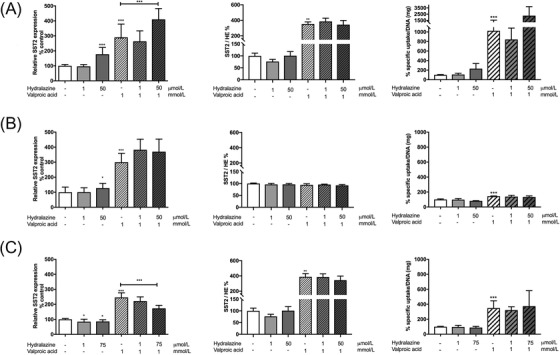
Effect of epigenetic treatment with valproic acid and hydralazine on the human neuroendocrine tumor cell lines (A) BON‐1, (B) GOT1 and (C) NCI‐H727. Graphs show somatostatin receptor subtype 2 (SST_2_) mRNA expression levels, SST_2_ protein levels and uptake of radiolabelled ^111^In‐DOTATATE as percentage increase or decrease compared to control cells. DNA quantification (as a measure for cell amount in cell growth experiments) was performed with Hoechst 33256 for BON‐1 and NCI‐H727, whereas Quant‐iT PicoGreen dsDNA reagent (Invitrogen, Breda, The Netherlands) was used for GOT1. For mRNA‐analysis TaqMan Universal PCR Master Mix (Applied Biosystems, Breda, The Netherlands) supplemented with primers and probes was used. SST_2_ expression was determined relative to three housekeeping genes (*GUSB*, *HPRT1* and *ACTB*) using the QuantStudio 7 Flex RT‐qPCR system with QuantStudio Real‐Time PCR software v1.5. Immunohistochemistry was performed using rabbit monoclonal anti‐SST_2_ IgG (NB‐49‐015, 1:25 dilution, NeoBiotech, Nanterre, France). Stained cells were visualized with the NanoZoomer 2.0 HT (Hamamatsu Photonics, Hamamatsu City, Japan) and SST_2_ staining intensity per cell was assessed using the CellProfiler software (version 4.0.7, www.cellprofiler.org). Internalization studies were performed with ^111^In‐DOTATATE. ^111^InCl_3_ (Curium Pharma, Petten, The Netherlands) was used to radiolabel DOTATATE (Bachem AG, Bubendorf, Switzerland) with a molar activity of 50 MBq/nmol. Data are shown as mean with the standard deviation of three (mRNA expression levels and radiolabelled ^111^In‐DOTATATE uptake) or two (immunohistochemistry) independent experiments. Data were normalized to control values, all set at 100%. HE, hematoxylin eosin. *p < .05, **p < .01, ***p < .001 according to one‐way ANOVA analysis with Tukey post hoc test after log transformation of data

Our study shows, for the first time, that contrary to the promising in vitro and in vivo data on epigenetic upregulation of SST_2_ expression, epigenetic treatment did not translate into the stimulation of ^68^Ga‐DOTATATE uptake in NET patients with low baseline SST expression.

This appears to be in contrast to the study with five SST‐positive patients who received vorinostat‐treatment for 4 days,^5^ but their observed change in the maximum standard uptake value (SUV_max_) of 1.3 could lack clinical relevance. Combined these studies might imply that either epigenetic upregulation of SST_2_ expression is only effective in patients with sufficient baseline ^68^Ga‐DOTATATE uptake, or the epigenetic effect depends on the epidrugs used or the drug levels achieved in patients are not sufficient to induce upregulation. The importance of choice and dosage of the epidrugs was shown by the effect of the DNMT inhibitor hydralazine, exhibiting only mild effects in pharmacologically unreachable dosages despite good efficiency observed in other tumours.^7^, ^8^ A possible future limitation for epigenetic treatment in NETs could also be the observed non‐specific effect of increased renal uptake in our patients. Although changes in uptake measures of up to 25% SUV_max_ between two scans have been described,^9^ the increase in renal uptake was seen in 78% of our patients in the second PET/CT. As all patients underwent the same hydration protocol before the scan and no changes in kidney function were noted, this could signify that the epigenetic treatment is not tumour specific and also activates basal expression of SST_2_ in renal tissue.^10^

In conclusion, short‐term epigenetic treatment with valproic acid and hydralazine had no stimulating effect on ^68^Ga‐DOTATATE uptake in nine patients with well‐differentiated NETs of various origins with low baseline SST expression, contradicting preclinical findings. Clinical trials with alternative epigenetic drugs or in patients with positive baseline SST_2_ expression may be able to clarify whether epigenetic treatment has a role in the treatment of NETs; however, a potential increase in renal uptake should be closely monitored.

## FUNDING INFORMATION

This study was supported by the Erasmus MC Foundation. JR was supported by a grant from the Swiss National Science Foundation (P2BSP3‐181720).

## CONFLICT OF INTEREST

RF received research support from Ipsen, Strongbridge and Corcept as well as speaker fees from HRA Pharma, Novartis, Ipsen. WWDH received research support from AAA‐Novartis, speaker fees from Ipsen and AAA‐Novartis and is on the advisory board of AAA. TB received speaker fees, research support from AAA and is on the advisory board of AAA. JH received speaker fees from Ipsen and is on the advisory board of Novartis.

All other authors have no conflicts of interest to declare.

## Supporting information



Supporting Information
