# Supplementary material for: Effect of epigenetic treatment on SST2 expression in neuroendocrine tumour patients
Source: Clin Transl Med. 2022 Jul 22;12(7):e957. doi: 10.1002/ctm2.957 (PMC9304941; doi:10.1002/ctm2.957)
Supplement: Supplementary file 1 — Supporting Information [file CTM2-12-e957-s001.docx]

**SUPPLEMENTARY APPENDIX of the Letter to the Editor:**

**Effect of Epigenetic Treatment on SST_2_ Expression in Neuroendocrine Tumor Patients**

Julie Refardt^1,2^, Maria J. Klomp^1,3^, Peter M. van Koetsveld^1^, Fadime Dogan^1^, Mark Konijnenberg^3^, Tessa Brabander^3^, Richard A. Feelders^1^, Wouter W. de Herder^1^, Leo J. Hofland^1^, Johannes Hofland^1^

***^1^****ENETS Center of Excellence, Department of Internal Medicine, Section of Endocrinology, Erasmus Medical Center, Rotterdam, The Netherlands; ^2^ENETS Center of Excellence, Department of Endocrinology, University Hospital Basel, Basel, Switzerland;* ***^3^****ENETS Center of Excellence, Department of Radiology & Nuclear Medicine, Erasmus Medical Center, Rotterdam, The Netherlands;*

**Corresponding author:**

Dr. Julie Refardt

ENETS Center of Excellence

Department of Endocrinology, University Hospital Basel

Petersgraben 4, 4031 Basel, Switzerland

[Julie.refardt@usb.ch](mailto:Julie.refardt@usb.ch)

**TABLE OF CONTENTS**

**METHODS** page 3

1. **Clinical study** page 3

**Study design and participants** page 3

**Study procedures and assessments** page 3

**Imaging and radiological assessment** page 4

1. ***In vitro* experiments** page 4

**Cell culture** page 4

**Epigenetic treatment and evaluation** page 4

1. **Statistical analysis** page 6

**RESULTS** page 7

**Safety** page 7

**Cell line experiments** page 7

Basal SST_2_ expression, SST_2_ protein levels and
^111^In-DOTATATE uptake page 7

**Supplementary Figure S1** page 8

**Supplementary Figure S2** page 9

**References**  page 10

**METHODS**

1. ***Clinical study***

***Study design and participants***

This prospective proof-of-concept study was performed at the Erasmus Medical Center Rotterdam, the Netherlands from 07/2019 until 06/2021. Eligible patients were ≥18 years of age, had an inoperable or metastatic NET with well-differentiated histology grade 1, 2 or 3 and low SST uptake on ^68^Ga-DOTATATE PET scan. Patients with hypotension (systolic blood pressure <90 mmHg), heart failure NYHA III-IV, creatinine clearance <50 ml/min, liver transaminases >3 times upper normal range, uncontrolled hormonal symptoms including severe diarrhea, serum albumin concentration <25 g/L, epilepsy or existing drug treatment which could not be stopped and interacted with the study medication were excluded.

***Study procedures and assessments***

Patients were recruited from the NET clinic at the ENETS Center of Excellence, Erasmus MC in Rotterdam. After signing the informed consent, screening included a medical questionnaire, physical examination and blood sampling. If ^68^Ga-DOTATATE PET/CT had been performed in a different institute or >3 months before inclusion, it was repeated during screening.

The primary endpoint was the percentage of patients with an increase in uptake of ≥1 point ^68^Ga-DOTATATE in the tumor lesions according to a predefined uptake scale. Grade 1 uptake was below the liver, grade 2 similar to the liver, grade 3 higher than the liver and grade 4 higher than uptake in spleen/kidneys. Pre-specified secondary endpoints included the change in tumoral ^68^Ga-DOTATATE uptake as well as physiological uptake of liver, kidneys and spleen as measured by peak standardized uptake value (SUV), and impact of epigenetic treatment on clinical and laboratory parameters.

Adverse events were registered according to Common Terminology Criteria for Adverse Events (CTCAE) version 5.0.

***Imaging and radiological assessment***

^68^Ga-DOTATATE was prepared locally in our institute. PET images were acquired on a Siemens Biograph mCT PET/CT scanner (Siemens Healthineers, Erlangen, Germany).

Quantitative assessment of lesions and physiological uptake was performed on Hermes Hybrid Viewer software (V 2.6D Hermes medical solutions, Stockholm) software.

1. ***In vitro experiments with NET cell lines***

For the cell line experiments, the human pancreatic NET cell line BON-1 (kind gift of Dr. Townsend, University of Texas, Medical branch, Galveston, TX, USA), the human midgut NET cell line GOT1 (kind gift of Ola Nilsson, Sahlgrenska Cancer Center, University of Gothenburg, Sweden) and the human pulmonary carcinoid cell line NCI-H727 (ATCC CRL-5815) were used.

***Cell Culture***

BON-1 cells were cultured in DMEM/F-12 (1:1) supplemented with 10% (v/v) FCS, 2 mM L-glutamine, 1.25 mg/L fungizone, and 100 U/ml penicillin; GOT-1 cells were cultured in RPMI medium 1640 supplemented with 10% (v/v) FCS, 2 mM L-glutamine, 100 U/mL penicillin, 100 µg/mL streptomycin, 1.0 g/L insulin, 0.55 g/L transferrin, and 67 µg/L selenite; NCI-H727 cells were cultured in RPMI medium 1640 + L-glutamine supplemented with 10% (v/v) FCS, 100 U/mL penicillin, and 100 µg/mL streptomycin. Once a week, BON-1 and NCI-H727 cells were trypsinized using 0.05% (v/v) trypsin + 0.53 mM EDTA and fresh medium was added on day four. GOT1 cells were trypsinized every two weeks using 0.05% (v/v) trypsin + 0.53 mM EDTA supplemented with DNAse (2 U/mL) with medium refreshment after one week.

***Epigenetic treatment and evaluation***

Valproic acid sodium salt (VPA; Sigma-Aldrich, Zwijndrecht, The Netherlands) and hydralazine (Hydralazine HCl; Selleckchem.com) were dissolved in the according cell line culture media. Dose-response studies were performed based on a 7-day treatment schedule. One day before the start of the epigenetic treatment, cells were plated in 24-well plates.

Epigenetic treatment: Cells were plated in T75 flasks on day zero. VPA and hydralazine, alone or in combination, were added on day 1 at their IC_50_ growth inhibitory concentrations and at the maximum treatment dosage used in patients (equal to IC_50_ dosage for VPA, lower dosage for hydralazine). Medium without or with drugs was refreshed on day 3. On day 5, cells were trypsinized and plated for further analysis. Exactly 4 hours after cell plating, the drugs were added again. On day 7, samples were collected for RT-qPCR analysis (24-well plates) and for internalization studies (12-well plates). For immunohistochemistry, cells were plated in chamber slides after pre-treatment with poly-L-lysine.

mRNA-Analysis: After lysis, cells were incubated with oligo(dT)_25_ dynabeads (Invitrogen, Breda, The Netherlands) to isolate poly-A+ mRNA, as described previously^4^. 23 µL H_2_O was added for elution, and 10 µL poly-A+ mRNA was used in the next steps. Poly-A+ mRNA was converted into cDNA using the commercial RevertAid First Strand cDNA synthesis kit (Thermo Scientific, Breda, The Netherlands). cDNA was also prepared without the addition of RevertAid Reverse Transcriptase to exclude DNA contamination. Samples were diluted by adding 180 µL H_2_O. Afterwards 5 µL sample was mixed with 7.5 µL Taqman Universal PCR mastermix (Applied Biosystems, Breda, The Netherlands) supplemented with primers and probes. SST_2_ expression was determined relative to three housekeeping genes (HKGs). For analysis, the QuantStudio 7 Flex RT-qPCR system with QuantStudio Real-Time PCR software v1.5 was used. The number of copies for SST_2_ and all HKGs was calculated by the efficiency factor to the power of ∆Ct (i.e., 40 minus measured Ct). Subsequently, the relative SST_2_ expression was calculated by dividing the number of SST_2_ copies by the geometric mean of all HKGs.

Immunohistochemistry: Cells were fixed with 4% paraformaldehyde for 20 minutes, before incubating them with 50% methanol for 3 minutes and 100% methanol for 3 minutes. Then, cells were permeabilized (0.1% triton X100 detergent in 1x PBS) for 15 minutes, and blocked (1% BSA) for 1 hour at room temperature (RT). Rabbit monoclonal anti-SST2 IgG (NB-49-015, 1:25 dilution, NeoBiotech, Nanterre, France) was added (overnight, 4° C). Finally, the cells were incubated for 30 min at RT with HRP/anti-Rabbit/Mouse (Dako Detection System). Bound antibodies were visualized by incubation with freshly prepared DAB (Dako Detection System). For negative controls, primary antibody was omitted. Slides were counterstained with hematoxylin and mounted. Five locations per slide were used to assess the SST_2_ staining intensity per cell, using a 10x magnification and the CellProfiler software (version 4.0.7, www.cellprofiler.org).

^111^In-DOTATATE radiolabeling and internalization studies: DOTATATE (Bachem AG, Bubendorf, Switzerland) was radiolabeled with ^111^InCl_3_ (Curium Pharma, Petten, The Netherlands) as previously described^5^. Internalization studies were performed as previously described^6^. Cells were incubated with internalization medium (DMEM (1x)–GlutaMAX-I, 1% (wt/v) BSA, and 20 mM HEPES (pH 7.4)) supplemented with 10^-9^ M ^111^In-DOTATATE (50 MBq/nmol), with or without 10^-6^ M unlabeled DOTATATE, for 4 hours. Following incubation, the excess of unbound radiotracer was removed, and the membrane-bound and internalized radioactivity were determined. The protocol was adjusted for GOT1 cells due to insufficient cell adherence and included the collection of non-adherent cells (pelleted by centrifugation). For GOT1 cells, the total uptake was determined. Cell pellets of additional wells were collected and DNA content was measured as described above, to correct for possible differences in cell numbers.

1. ***Statistical analysis***

This proof-of concept clinical trial aimed to include 10 patients. Twelve patients were enrolled in the study. Two patients failed screening because of sufficient uptake on ^68^Ga-DOTATATE PET and other tumor diagnosis than NET, respectively. Ten patients started study treatment, but one patient withdrew from the study after one week due to adverse events.

Descriptive statistics were used to characterize clinical, laboratory and radiological data, summarized by median and inter-quartile-range (IQR) or mean and standard deviation (SD), categorical variables by frequency and percentages.

Statistical analyses were performed using SPSS version 25.0 (IBM Corp., Armonk, NY) and GraphPad Prism7. A two-sided significance level of 0.05 was set for every analysis.

**RESULTS**

***Safety***

A total of 18 adverse events occurred during the observation phase in ten patients of the intended to treat analysis set of which 14 were judged to be related to the study intervention. Five events were known effects of valproic acid treatment and involved neurocognitive symptoms, tiredness and/or nausea. Five other adverse events were classified as hydralazine-related and involved palpitations, hypotonia and/or water retention. One patient developed an exanthema and one suffered from glucose dysregulation, requiring adjustment of insulin treatment. The patient who stopped treatment due to side effects suffered from nausea with vomitus, headaches and generalized aches.

***Cell line experiments***

***Basal SST_2_ expression, SST_2_ protein levels and ^111^In-DOTATATE uptake***

BON-1 cells showed the lowest mean (SD) SST_2_ expression level of 0.0046 (0.001) (corrected for the geometric mean of three HKGs), followed by the NCI-H727 cells (0.0054 (0.001)) with the highest levels being measured in the GOT1 cells (0.142 (0.025)). Mean (SD) SST_2_ staining intensity per cell was also lowest for BON-1 cells with 7.95 (7.61), again followed by the NCI-H727 cells with 13.43 (4.72) and showing the highest levels for GOT1 cells with 91.83 (6.89), *Figure S2*. A similar trend was observed for the mean (SD) uptake of ^111^In-DOTATATE, which was 4.23 (1.86), 21.51 (6.09) and 327.0 (20.95) % added dose per milligram DNA for the three cell lines, respectively.

**Supplementary Figure S1**

**
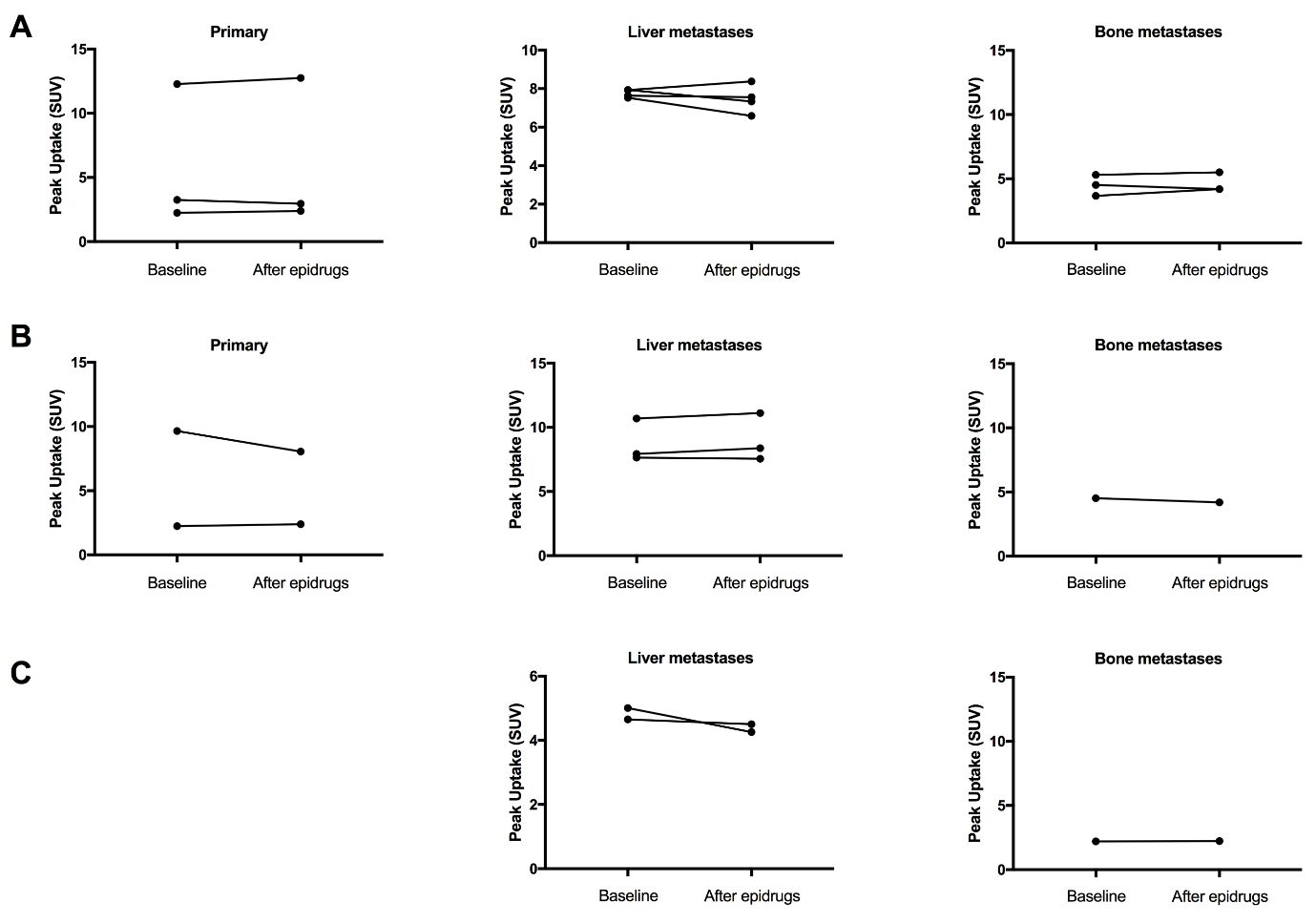
**

**Supplementary Figure S1**

**Figure 1** Change in peak uptake of ^68^Ga-DOTATATE on PET/CT at baseline and after 2-week epigenetic treatment in patients with neuroendocrine tumors with low somatostatin receptor expression according to their origin: A) lung, B) pancreas, C) small-intestine.

**Supplementary Figure S2**


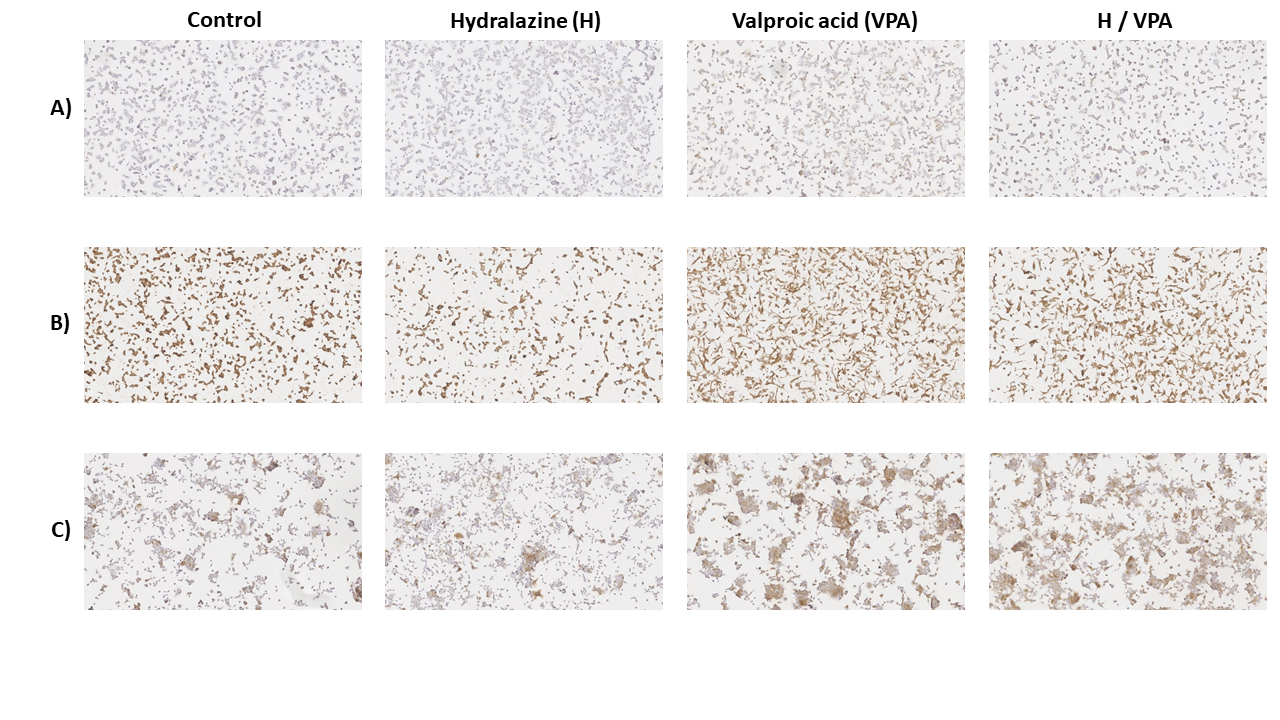


**Supplementary Figure S2** Immunohistochemistry of somatostatin receptor subtype 2 in

A) BON-1, B) GOT1, C) NCI-H727 shown in an untreated control group (control) and following epigenetic treatment with hydralazine (50 µmol/L), valproic acid (1 mmol/L) or the combined treatment of hydralazine / valproic acid (H / VPA).

Pictures were taken at 100x magnification.

**REFERENCES**

1. Gottlicher M, Minucci S, Zhu P, et al. Valproic acid defines a novel class of HDAC inhibitors inducing differentiation of transformed cells. *EMBO J*. Dec 17 2001;20(24):6969-78. doi:10.1093/emboj/20.24.6969

2. Duenas-Gonzalez A, Coronel J, Cetina L, Gonzalez-Fierro A, Chavez-Blanco A, Taja-Chayeb L. Hydralazine-valproate: a repositioned drug combination for the epigenetic therapy of cancer. *Expert Opin Drug Metab Toxicol*. Oct 2014;10(10):1433-44. doi:10.1517/17425255.2014.947263

3. Veenstra M, van Koetsveld P, Dogan F, et al. *Epidrug-induced upregulation of functional somatostatin type 2 receptors in human pancreatic neuroendocrine tumor cells*. 2016.

4. Klomp MJ, Dalm SU, van Koetsveld PM, Dogan F, de Jong M, Hofland LJ. Comparing the Effect of Multiple Histone Deacetylase Inhibitors on SSTR2 Expression and [(111)In]In-DOTATATE Uptake in NET Cells. *Cancers (Basel)*. Sep 29 2021;13(19)doi:10.3390/cancers13194905

5. de Blois E, Chan HS, de Zanger R, Konijnenberg M, Breeman WA. Application of single-vial ready-for-use formulation of 111In- or 177Lu-labelled somatostatin analogs. *Appl Radiat Isot*. Feb 2014;85:28-33. doi:10.1016/j.apradiso.2013.10.023

6. Dalm SU, Nonnekens J, Doeswijk GN, et al. Comparison of the Therapeutic Response to Treatment with a 177Lu-Labeled Somatostatin Receptor Agonist and Antagonist in Preclinical Models. *J Nucl Med*. Feb 2016;57(2):260-5. doi:10.2967/jnumed.115.167007
